# Supplementary material for: Integrative structural analysis of NF45–NF90 heterodimers reveals architectural rearrangements and oligomerization on binding dsRNA
Source: Nucleic Acids Res. 2025 Mar 28;53(6):gkaf204. doi: 10.1093/nar/gkaf204 (PMC11952958; doi:10.1093/nar/gkaf204)
Supplement: gkaf204_Supplemental_Files [file gkaf204_supplemental_files.zip › Supplementary_tables1-2_and_figures_1-5.pdf]

Supplementary tables and figures for

Integrative structural analysis of NF45-NF90 heterodimers reveals architectural rearrangements and oligomerisation on binding dsRNA.

Sophie Winterbourne, Uma Jayachandran, Juan Zou, Juri Rappsilber, Sander Granneman and Atlanta G. Cook

Supplementary Table 1. Oligonucleotides used in this study

Name	RNA/ DNA	Sequence 5′->3′
18 bp top strand for EDC	RNA	UCACUUUCAUAAUGCUGG (Uniform 2′ Fluoro)
18 bp bottom strand for EDC	RNA	CCAGCAUUAUGAAAGUGA
25 bp GC-rich top strand for CLMS	RNA	GCCGCGGAGGCCCCGCCGUGGGCCC
25 bp GC-rich bottom strand for CLMS	RNA	GGGCCCACGGCGGGGCCUCCGCGGC
25 bp for SAXS top strand	RNA	UCACUUUCAUAAUGCUGGUCACUUU
25 bp for SAXS bottom strand	RNA	AUGCUGGUCACUUUCAUAAUGCUGG
36 bp for SAXS top strand	RNA	UCACUUUCAUAAUGCUGGUCACUUUCAUAAUGCUGG
36 bp for SAXS bottom strand	RNA	CCAGCAUUAUGAAAGUGACCAGCAUUAUGAAAGUGA
54 bp for SAXS top strand	RNA	UCACUUUCAUAAUGCUGGUCACUUUCAUAAUGCUGGUCACUUUCAUAAUGCUGG
54 bp for SAXS bottom strand	RNA	CCAGCAUUAUGAAAGUGACCAGCAUUAUGAAAGUGACCAGCAUUAUGAAAGUGA
**PCR primers**		
310bp_Cyp1A1_F	DNA	5′ P aatgccgttttattccgatttc
310bp_Cyp1A1_R	DNA	5′ P gttattgaagttcccggacac

Supplementary Table 2.

(a) Sample details: SAXS experimental details and data parameters for NF90-NF45 constructs					
Sample	$NF90_{long}$-NF45		$NF90_{DZF}$-$NF45_{DZF}$		$NF90_{dsRBDs}$
	NF90	NF45	NF90	NF45	NF90
Organism	*Mus musculus*	*Homo sapiens*	*Mus musculus*	*Mus musculus*	*Mus musculus*
Source (Catalogue No. or reference)	*E. coli* expressed	*E. coli* expressed	*E. coli* expressed	*E. coli* expressed	*E. coli* expressed
Uniprot ID (residues in construct) + uncleaved tag	Q9Z1X4 (1-591) + Additional G + LE scar site + HHHHHH-tag at C-terminus	Q12905 (1-390) + GST-tag (GPLGSPEF) at N-terminus	Q9Z1X4 1-381 + GH TEV cleavage scar at N-terminus	Q9CXY6 29-390 + GS at N-terminus from precision site	Q9Z1X4 395-592 + Additional G at N-terminus from GST-tag
Molecular mass M from chemical composition (Da)	65897	43846	42468	40320	21138
Column	SEC-SAXS column, s200 increase 3.2/200				
Loading concentration (mg/ml)	7.75		10		10
Injection volume (µl)	60		60		60
Flow rate (ml/min)	0.1		0.1		0.1
Solvent composition	20 mM HEPES pH 7.5 150 mM NaCl 1 mM DTT				

(b) SEC-SAXS data collect parameters	
Instrument	Diamond Light Source Ltd Synchrotron, BL21 beamline, EigerX 4M detector (Dectris)
Source	Bending magnet
Wavelength (Å)	0.954
Beam size at focal point (µm)	
Sample-to-detector distance (m)	3.7
q-measurement range ($Å^{-1}$)	
Exposure time	Continuous 0.005 s data-frame measurements of SEC elution (915 frames)
Sample temperature (°C)	15

(c) Software employed for SAXS data reduction, analysis and interpretation	
Sample – Solvent subtraction	ScÅtter IV (https://bl1231.als.lbl.gov/scatter/)
Basic analyses: Guinier, P(r), V_p	ScÅtter IV (https://bl1231.als.lbl.gov/scatter/)
Shape/bead modelling	GASBOR 2.3i (Svergun et al., 2001)
Atomic structure modelling	MultiFoXS (Schneidman-Duhovny et al., 2016) via web server (https://modbase.compbio.ucsf.edu/multifoxs/)
Molecular graphics	PyMOL 2.5.4

(d) Structural parameters			
	$NF90_{long}$-NF45	$NF90_{DZF}$-$NF45_{DZF}$	$NF90_{dsRBDs}$
I(0) (cm^{-1}) [from Guinier]	0.0894	0.119	0.0346
R_g (Å) [from Guinier]	42.53 ± 0.177	36.08 ± 0.6563	35.82 ± 0.8592

qminRg - qmaxRg used for Guinier	0.6692 - 1.1881	0.7444 - 1.2842	0.7024 - 1.0828
Score	1.183	1.076	1.043
I(0) (cm^{-1}) [from p(r)]	0.101	0.119	0.0309
R_g (Å) [from p(r)]	46.93	35.93	34.68
D_{max} (Å) [from p(r)]	171	125.5	126
Porod volume, Vp ($Å^{-3}$)	270388	167777	48622
Volume-of-correlation, Vc	821.9	590.5	314.1
Molecular mass (kDa) [from Vp]	162.9	101.1	29.3
Molecular mass (kDa) [from Vc]	116.8	80.2	22.8

(e) Shape modelling results

	$NF90_{long}$-NF45	$NF90_{DZF}$-$NF45_{DZF}$	$NF90_{dsRBDs}$
GASBOR (default parameters)			
Number of dummy residues	998	747	199
Symmetry/anisotropy assumptions	P1, none	P1, none	P1, none
χ^2 value	1.413	1.079	1.301

(f) Atomistic modelling

Multistate/ensemble models

MultiFoXS (10 000 models in starting set)

	$NF90_{long}$-NF45	$NF90_{DZF}$-$NF45_{DZF}$	$NF90_{dsRBDs}$
Starting crystal structures	4AT7, 5DV7, AF-Q9Z1X4, AF-Q9CXY6	4AT7, 5DV7, AF-Q9Z1X4, AF-Q9CXY6	4AT7, 5DV7, AF-Q9Z1X4, AF-Q9CXY6
Flexible residues	1-28A, 362-390A, 1-5B, 55-86B, 341-353B, 376-381B, 468-518B, 591B	362-390A, 1-5B, 55-86B, 341-353B, 376-381B	394-402A, 468-518A
No. of states	4	1	4
χ^2 CORMAP p-values	1.18	1.23	1.04
c1, c2	1.00, 1.03	1.02, 0.35	1.03, 1.80
Rg values of each state (Å)	1) 46.74	1) 35.47	1) 31.27
	2) 47.97		2) 42.23
	3) 54.78		3) 56.66
	4) 39.51		4) 28.59
Weights wn	w1: 0.095	w1: 1.0	w1: 0.553
	w2: 0.587		w2: 0.314
	w3: 0.246		w3: 0.08
	w4: 0.072		w4: 0.052

(g) Data and model deposition IDs

	$NF90_{long}$-NF45	$NF90_{DZF}$-$NF45_{DZF}$	$NF90_{dsRBDs}$
SASBDB	SASDUD5	SASDUB5	SASDUA5

(a) Sample details: SAXS experimental details and data parameters for NF45-NF90$_{long}$ with dsRNA

	Organism	Source	UniProt ID (residues in construct) + uncleaved tag	Molecular mass (Da)
NF45	*Homo sapiens*	*E. coli* expressed	Q12905 (1-390) + GST-tag (GPLGSPEF) at N-terminus	43846
NF90	*Mus musculus*	*E. coli* expressed	Q9Z1X4 (1-591) + Additional G + LE scar site with HHHHHH-tag at C-terminus	65897
25mer	-	Biomers RNA oligos	5’ UCA CUU UCA UAA UGC UGG UCA CUU U 3’<br>3’ CCA GCA UUA UGA AAG UGA CCA GCA U 5’	15949
36mer	-	Biomers RNA oligos	5’ UCA CUU UCA UAA UGC UGG UCA CUU UCA UAA UGC UGG 3’<br>3’ CCA GCA UUA UGA AAG UGA CCA GCA UUA UGA AAG UGA 5’	23102
54mer	-	Biomers RNA oligos	5’ UCA CUU UCA UAA UGC UGG UCA CUU UCA UAA UGC UGG UCA CUU UCA UAA UGC UGG 3’<br>3’ CCA GCA UUA UGA AAG UGA CCA GCA UUA UGA AAG UGA CCA GCA UUA UGA AAG UGA 5’	65897

Samples	NF90-NF45	NF90-NF45 25mer 2:1	NF90-NF45 25mer 4:1	NF90-NF45 36mer 2:1	NF90-NF45 36mer 4:1	NF90-NF45 54mer 2:1	NF90-NF45 54mer 4:1	NF90-NF45 54mer 6:1

Column	SEC-SAXS column, s200 increase 3.2/200
Loading concentration (mg/ml)	5.5
Injection volume (µl)	60
Flow rate (ml/min)	0.075
Solvent composition	20 mM HEPES pH 7.5 150 mM NaCl 1 mM DTT

(b) SEC-SAXS data collection

Instrument	Diamond Light Source Ltd Synchrotron, BL21 beamline, EigerX 4M detector (Dectris)
Source	Bending magnet
Wavelength (Å)	0.9464
Beam size at focal point (µm)	50×50
Sample-to-detector distance (m)	3.7193
q-measurement range ($Å^{-1}$)	0.0045-0.34
Exposure time	Continuous 0.005 s data-frame measurements of SEC elution.
Frames	600
Sample temperature (°C)	15

(c) Software employed for SAXS data reduction, analysis and interpretation

Sample – Solvent subtraction	Chromixs (Panjkovich and Svergun, 2018) from ATSAS 3.2.1 (Manalastas-Cantos et al., 2021)
Basic analyses: Guinier, P(r), V_p	ScÅtter IV (https://bl1231.als.lbl.gov/scatter/)
Shape/bead modelling	DAMMIF (Franke and Svergun, 2009) and DAMMIN (Svergun, 1999) via ATSAS 3.2.1 (Manalastas-Cantos et al., 2021)
Atomic structure modelling	MultiFoXS (Schneidman-Duhovny et al., 2016) via web server (https://modbase.compbio.ucsf.edu/multifoxs/)
Molecular graphics	PyMOL 2.5.4

(d) Structural parameters

	NF90-NF45	NF90-NF45 25mer 2:1	NF90-NF45 25mer 4:1	NF90-NF45 36mer 2:1	NF90-NF45 36mer 4:1	NF90-NF45 54mer 2:1	NF90-NF45 54mer 4:1	NF90-NF45 54mer 6:1

I(0) (cm^{-1}) [from Guinier]	0.089	0.1905	0.08928	0.2563	0.2354	0.3006	0.4032	0.229
R_g (Å) [from Guinier]	48.03 ± 0.08089	58.95 ± 0.06254	58.12 ± 0.08977	63.90 ± 0.09438	63.62 ± 0.09487	73.86 ± 0.1347	79.29 ± 0.08955	79.47 ± 0.1291
$q_{min}R_g$ - $q_{max}R_g$ used for Guinier	0.6519 - 1.2150	0.8073 - 1.2314	0.6406 - 1.1960	0.8198 - 1.2037	0.7086 - 1.1893	1.0175 - 1.2406	0.7101 - 1.2339	0.7132 - 1.1685
Score	1.023	1.087	1.03	1.075	1.076	1.073	1.069	1.079
I(0) (cm^{-1}) [from p(r)]	0.0801	0.1714	0.08193	0.2324	0.2157	0.2676	0.3579	0.2069
R_g (Å) [from p(r)]	46.77	57.05	56.9	61.57	63.73	71.63	77.66	77.54
D_{max} (Å) [from p(r)]	171	198.5	204.5	212	220.5	250	264.5	260.5
Porod volume, V_p ($Å^{-3}$)	234456	398098	399715	587120	595567	613121	788402	791908
Volume-of-correlation, V_c	813.3	1278.9	1239.1	1461.2	1532.9	1759.4	2108.4	2009
Molecular mass (kDa) [from V_p]	141.2	239.8	240.8	353.7	358.8	369.4	474.9	477.1

(e) Shape modelling results

DAMMIF (default parameters, 10 repetitions), averaged with DAMAVER and refined with DAMMIN

	NF90-NF45	NF90-NF45 25mer 2:1	NF90-NF45 25mer 4:1	NF90-NF45 36mer 2:1	NF90-NF45 36mer 4:1	NF90-NF45 54mer 2:1	NF90-NF45 54mer 4:1	NF90-NF45 54mer 6:1
Symmetry/ anisotropy assumption	P1, none	P1, none	P1, none	P1, none	P1, none	P1, none	P1, none	P1, none
χ^2 value	1.274	1.4	1.067	1.309	1.354	1.288	1.201	1.491
Constant subtraction procedure	3.37×10^{-4}	6.60×10^{-4}	2.93×10^{-4}	1.09×10^{-3}	1.01×10^{-3}	1.96×10^{-3}	2.72×10^{-3}	1.50×10^{-3}
Model resolution (SASRES) (Å)	44.4151	56.0733	45.6861	61.2184	60.5901	69.8233	76.7173	68.5343

(f) Atomistic modelling	NF90-NF45
Multistate/ensemble models	MultiFoXS (10 000 models in starting set)
Starting crystal structures	4AT7, 5DV7, AF-Q9Z1X4, AF-Q9CXY6
Flexible residues	1-28A, 362-390A, 1-5B, 55-86B, 341-353B, 376-381B, 468-518B, 591B
No. of states	5
χ^2 CORMAP p-values	1.23
c1, c2	1.02, 0.31
Rg values of each state (Å)	1) 50.76, 2) 43.81, 3) 58.09, 4) 52.30, 5) 39.54
Weights wn	w1: 0.224, w2: 0.304, w3: 0.172, w4: 0.15, w5: 0.15

(g) Data and model deposition IDs

	NF90-NF45	NF90-NF45 25mer 2:1	NF90-NF45 25mer 4:1	NF90-NF45 36mer 2:1	NF90-NF45 36mer 4:1	NF90-NF45 54mer 2:1	NF90-NF45 54mer 4:1	NF90-NF45 54mer 6:1
SASBDB	SASDUC5	SASDUE5	SASDUF5	SASDUG5	SASDUH5	SASDUJ5	SASDUK5	SASDUL5

FRANKE, D. & SVERGUN, D. I. 2009. DAMMIF, a program for rapid ab-initio shape determination in small-angle scattering. *J Appl Crystallogr,* 42**,** 342-346.

MANALASTAS-CANTOS, K., KONAREV, P. V., HAJIZADEH, N. R., KIKHNEY, A. G., PETOUKHOV, M. V., MOLODENSKIY, D. S., PANJKOVICH, A., MERTENS, H. D. T., GRUZINOV, A., BORGES, C., JEFFRIES, C. M., SVERGUN, D. I. & FRANKE, D. 2021. ATSAS 3.0: expanded functionality and new tools for small-angle scattering data analysis. *J Appl Crystallogr,* 54**,** 343-355.

PANJKOVICH, A. & SVERGUN, D. I. 2018. CHROMIXS: automatic and interactive analysis of chromatography-coupled small-angle X-ray scattering data. *Bioinformatics,* 34**,** 1944-1946.

SCHNEIDMAN-DUHOVNY, D., HAMMEL, M., TAINER, J. A. & SALI, A. 2016. FoXS, FoXSDock and MultiFoXS: Single-state and multi-state structural modeling of proteins and their complexes based on SAXS profiles. *Nucleic Acids Research,* 44**,** W424-W429.

SVERGUN, D. I. 1999. Restoring low resolution structure of biological macromolecules from solution scattering using simulated annealing. *Biophys J,* 76**,** 2879-86.

SVERGUN, D. I., PETOUKHOV, M. V. & KOCH, M. H. 2001. Determination of domain structure of proteins from X-ray solution scattering. *Biophys J,* 80**,** 2946-53.

Figure S1

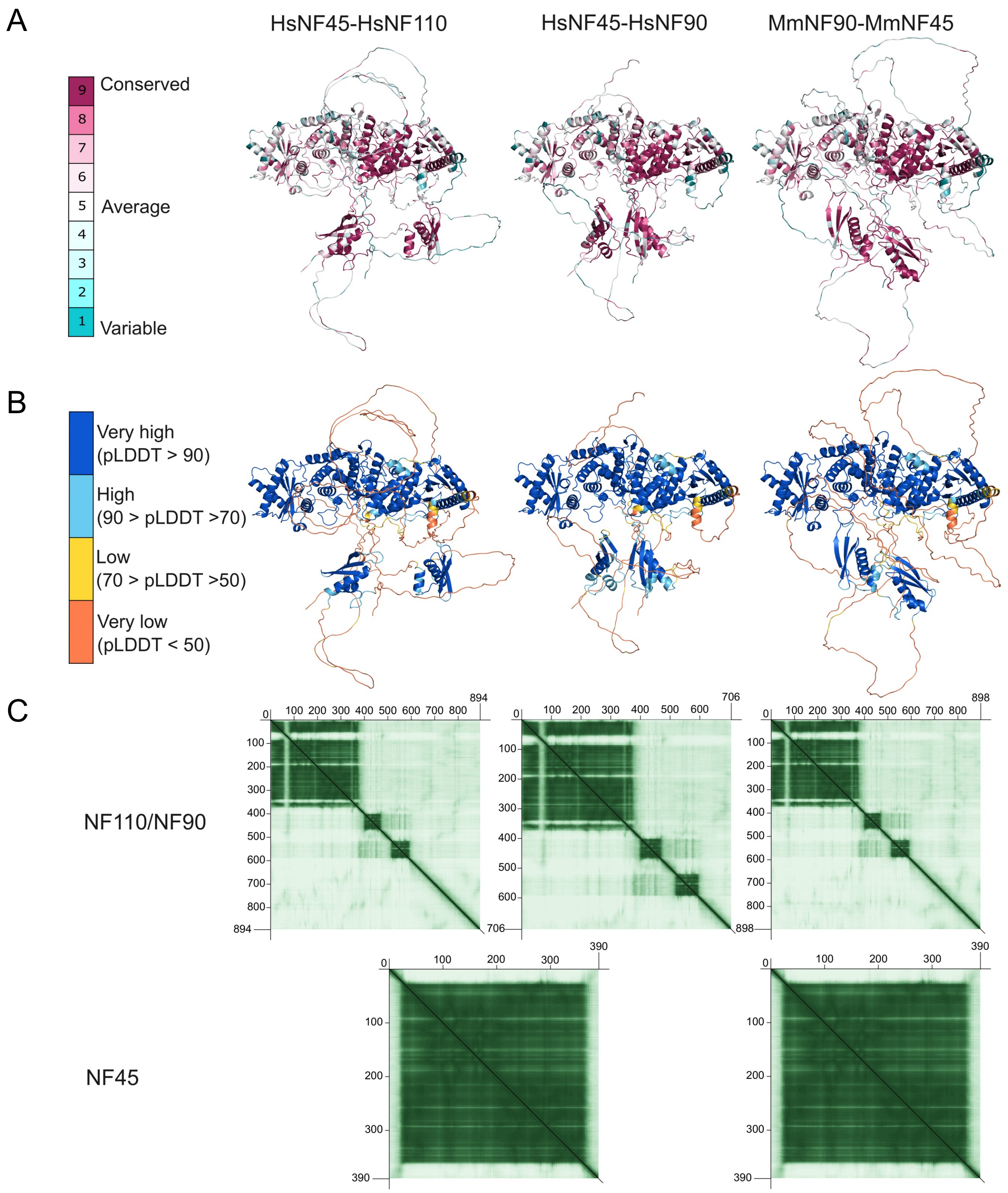

Figure S1. NF45-NF90 complexes reconstructed from AlphaFold2 models. (**A**) Reconstructed models showing coloring by ConSurf conservation score. (**B**) Models as shown in (**A**) coloured by pLDDT score. (**C**) Position aligned error matrix plots for HsNF110, HsNF90, MmNF90, HsNF45 and MmNF45. Darker green positions indicate predicted close distances within the model.

Figure S2

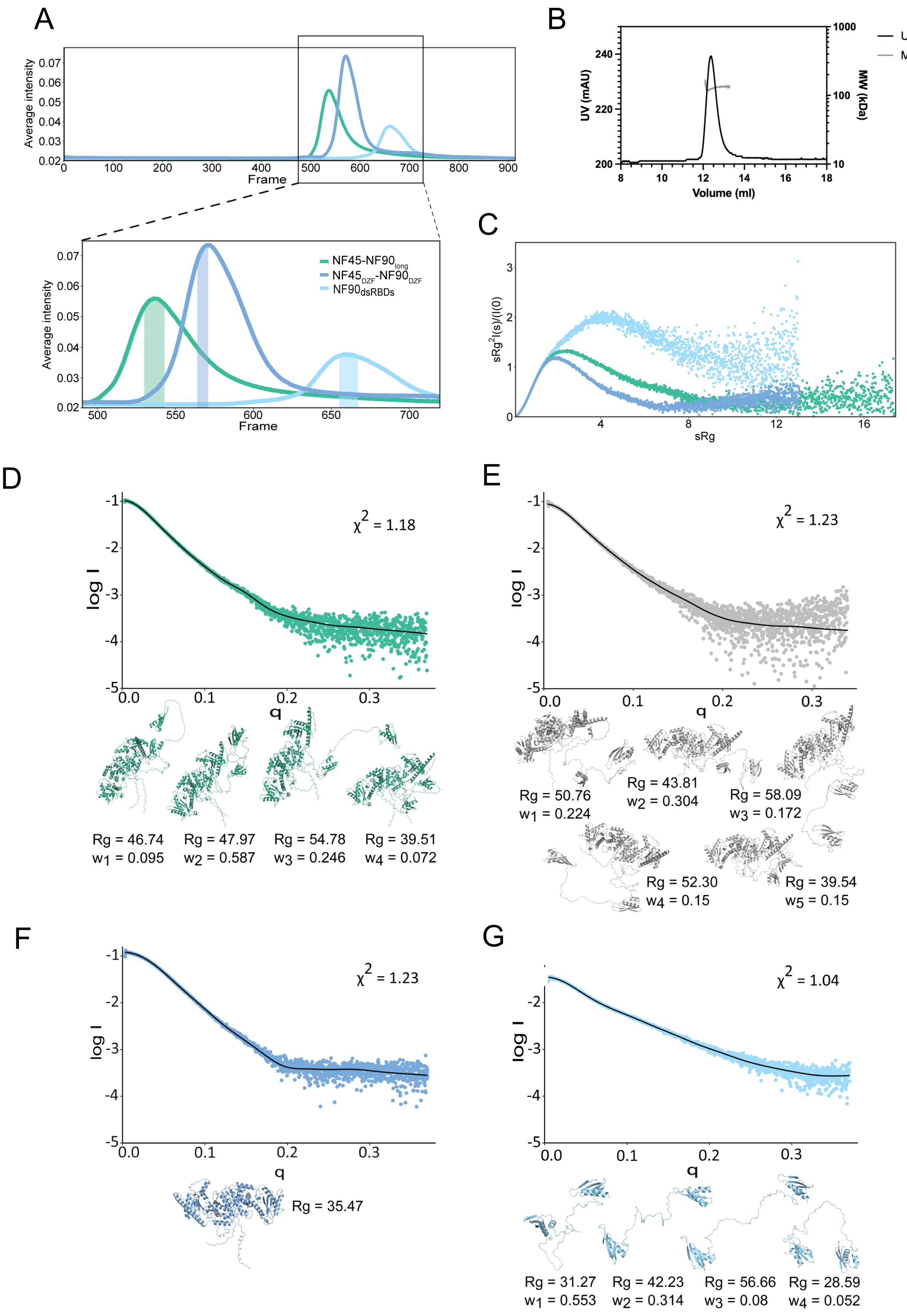

Figure S2. NF45-NF90 complexes in solution show compaction of domains. (**A**) SEC-SAXS profiles of three constructs with inset showing the data used for subsequent analysis. (**B**) SEC-MALS analysis of NF45-$NF90_{long}$. (**C**) Normalised Kratky plots for the three constructs analysed in (**A**). (**D-G**) Conformational diversity of NF45-NF90 is consistent with SAXS data. The fitting to SAXS data is given by the black line in each case. MultiFoXS analysis with (**D**) and (**E**) NF45-$NF90_{long}$ from two independent datasets, (**F**) $NF45_{DZF}$-$NF90_{DZF}$ with a single model, (**G**) $NF90_{dsRBDs}$ with four models.

Figure S3

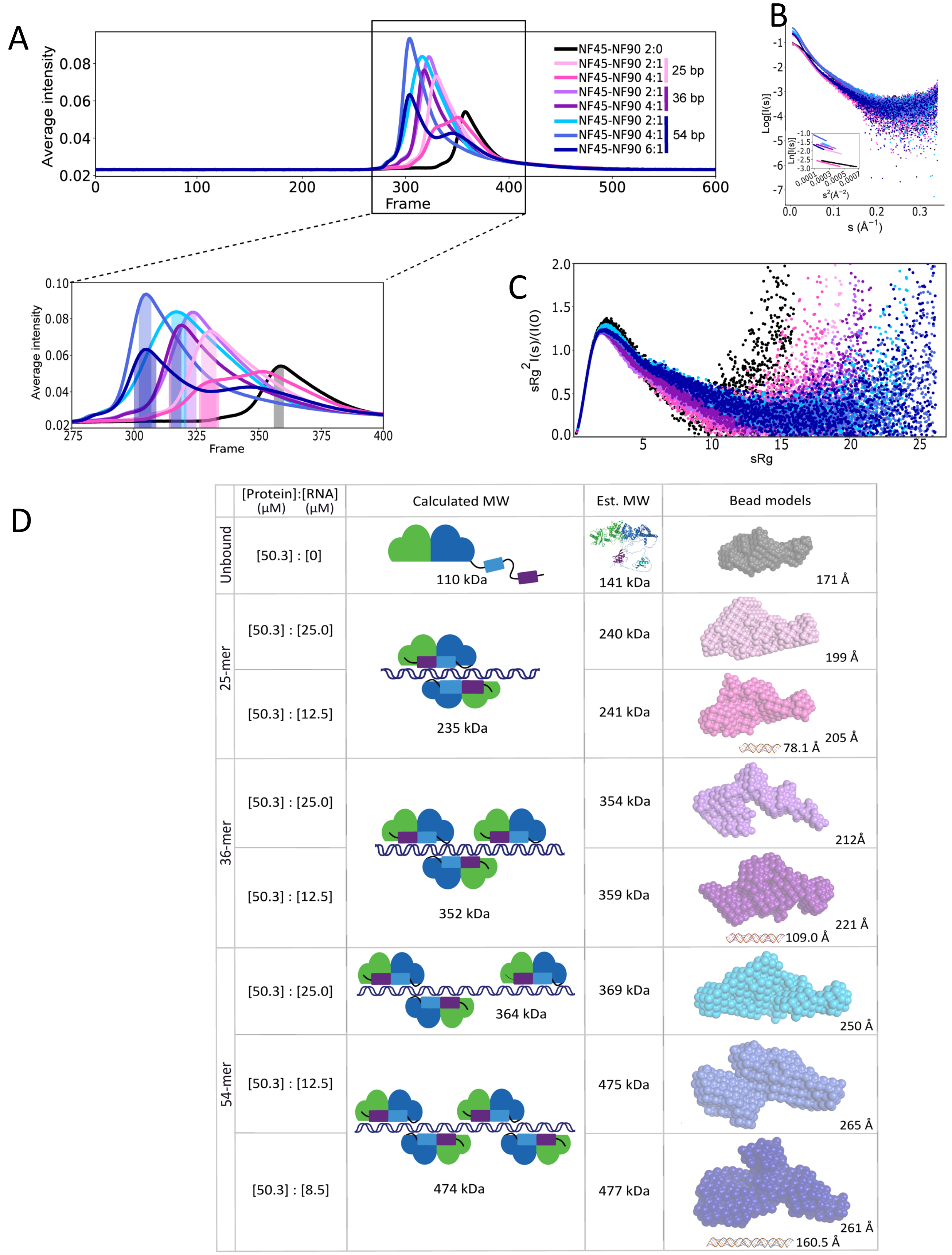

Figure S3. Solution analysis of NF45-NF90 binding to dsRNA of increasing lengths. Eight SAXS measurements were carried out with no dsRNA or with 25 bp, 36 bp and 54 bp RNA at increasing molar ratios of protein:dsRNA. (**A**) Intensity profiles of samples as eluted from size exclusion chromatography (Fig. 4A) with zoomed inset figures showing frames selected for real space analysis. (**B**) Extracted scattering curves for all samples with inset of Guinier analysis. (**C**) Normalised Kratky plots for all SAXS samples. (**D**) A summary of SAXS analysis. Molar ratios of complexes are shown in the first column. Likely molecular compositions with calculated molecular masses are shows as cartoon models in the centre, with SAXS-derived masses and bead models shown on the right as in Fig. 3B. D_{max} values are indicated under each model with an RNA model to scale.

Figure S4

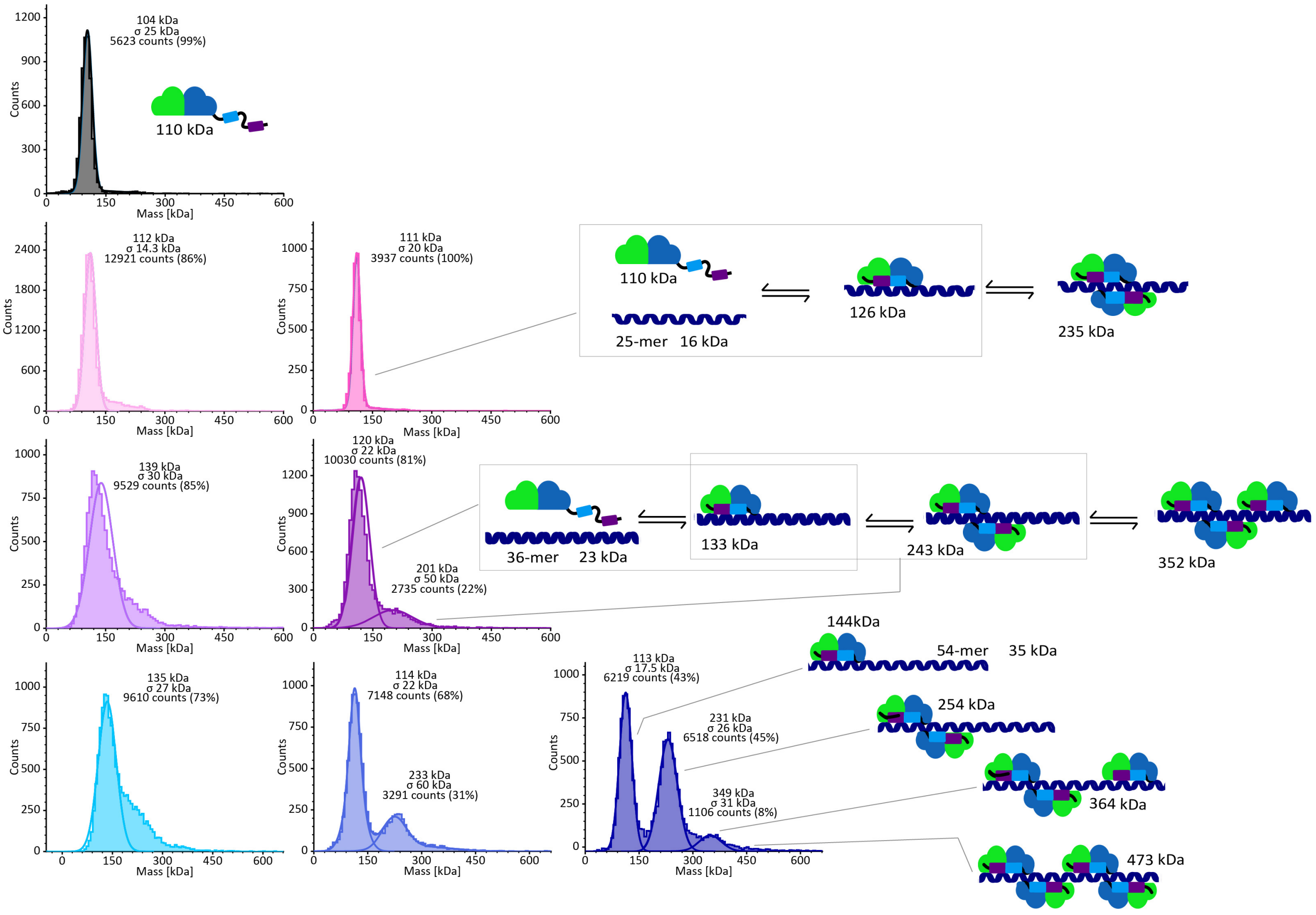

Figure S4: Population mass distributions of NF45-NF90$_{long}$ with increasing lengths of dsRNA by mass photometry. Masses given above peaks are median values, with associated standard deviations (σ), providing estimates of the molecular weight. Grey: NF45-NF90 complexes alone (109 kDa). Pink: NF45-NF90 in a 2:1 ratio and 4:1 ratio with 25 bp dsRNA; Purple: NF45-NF90 in a 2:1 and 4:1 ratio with 36 bp dsRNA; Blue: NF45-NF90 in a 2:1, 4:1 or 6:1 ratio with 54 bp dsRNA. Cartoons depict the likely equilibria and species present in each sample, with calculated molecular weights for those species.

Figure S5

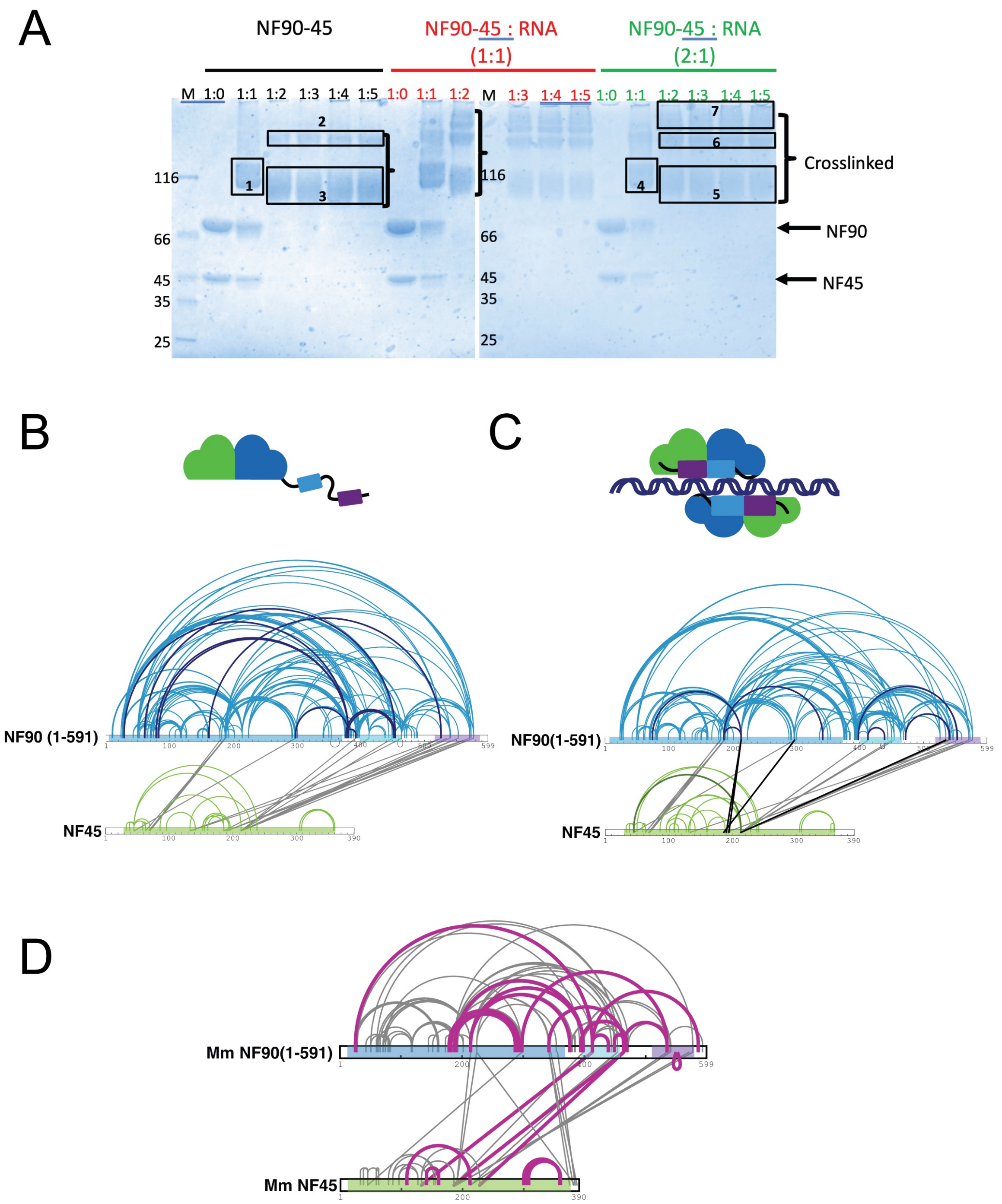

Figure S5. EDC crosslinking of NF45-NF90$_{long}$ complexes. (**A**) SDS-PAGE analysis of cross-linked samples, showing titration of EDC crosslinker with different samples of NF45-NF90$_{long}$. Boxes indicate the samples that were included in analysis. Samples 1-3 were used for data presented in Fig. 4A; Samples 4-6 were used for data present in Fig. 4B. (**B**) Data corresponding to Fig. 4C showing both quantified (dark) and non-quantified cross-links from this dataset. (**C**) Data corresponding to Fig. 4D showing both quantified (dark) and non-quantified cross-links from this dataset. (**D**) Data from all RNA bound samples including sample 7, showing cross-links specific to sample 7 in purple.

Figure S6

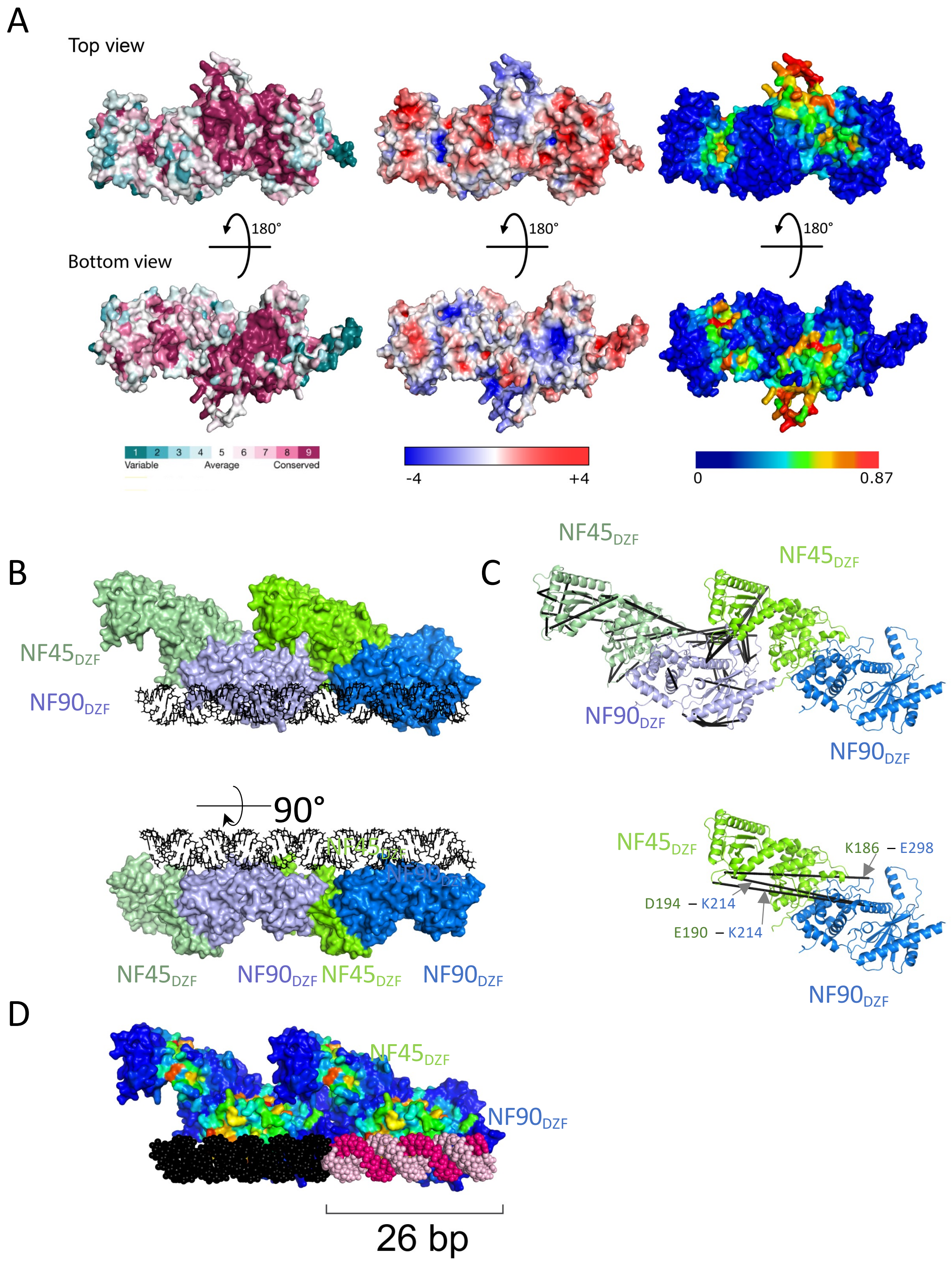

Figure S6. NF45-NF90$_{long}$ complexes oligomerize on long stretches of dsRNA. (**A**) Comparison of surface characteristics of NF45$_{DZF}$-NF90$_{DZF}$ showing conservation, electrostatics (-4 to +4 kTe) and RNA binding propensity (none to high). (**B**) Model shown in Fig. 5C but with surfaces colored by molecule. (**C**) Re-analysis of cross-links in the context of an open-ended oligomer model and displaying all measured cross-links. Bottom: NF45$_{DZF}$-NF90$_{DZF}$ heterodimer as shown in Fig. 5A, reproduced here for comparison with with top image. Crosslinks are shown as black lines. (**D**) Model as in (**B**) but highlighting RNA binding propensity and showing the dsRNA model as black or pink to indicate the length of 26 bp.
